# Cover crop species have contrasting influence upon soil structural genesis and microbial community phenotype

**DOI:** 10.1038/s41598-019-43937-6

**Published:** 2019-05-16

**Authors:** Aurelie Bacq-Labreuil, John Crawford, Sacha J. Mooney, Andrew L. Neal, Karl Ritz

**Affiliations:** 1 Division of Agriculture & Environmental Sciences, School of Biosciences, University of Nottingham, Sutton Bonington Campus, Leicestershire, LE12 5RD UK; 2 Department of Sustainable Agriculture Science, Rothamsted Research, West Common, Harpenden, AL5 2JQ UK

**Keywords:** Ecology, Plant physiology

## Abstract

Cover crops (plants grown in an agricultural rotation between cash crops) can significantly improve soil quality via sequestering carbon, retaining nutrients, decreasing soil erosion, and maintaining belowground biodiversity. However, little is known about the effects of such plants upon soil structure. The aim of the study was to assess the impact of four species typically used as cover crops and which have contrasting root architecture (*viz*. clover, black oat, phacelia, tillage radish) on soil structural genesis and the associated modification of microbial community structure in a clay soil. The four plant species were grown in a replicated pot experiment with sieved soil (<2 mm), with unplanted soil as control for 8 weeks. X-ray Computed Tomography was used to quantify the formation of pore networks in 3D and phospholipid fatty acid analysis was performed to characterise the microbial community phenotype. Black oats developed a greater soil-pore connectivity than the other species throughout the growth period, whereas phacelia decreased both the porosity and pore-connectivity. The microbial community phenotype under phacelia was notably different from the other species, with a greater proportion of fungal markers. Thus, different plant species have differential effects upon soil structural genesis and microbial community phenotype, which provides evidence that certain species may be more suitable as cover crops in terms of soil structural conditioning depending upon specific contexts.

## Introduction

Soil structure is an important factor affecting crop production, primarily due to the influence that the soil pore network exerts on root growth, soil fauna, nutrient, water and gas exchanges^1^. Soil quality can be defined as the capacity of a soil to deliver a requisite range of ecosystem goods and services, concomitant with that of air and water quality, and support human health and habitation^2,3^. Soil structure, being fundamental to soil function, is considered an effective indicator of soil quality^1^.

Moreover, plants are known to contribute to the structuring of soil via enmeshing and binding soil particles^4^ and to break down larger aggregates via root penetration^5^. The genesis of soil structure is a dynamic process that requires energy, which can be provided by plant roots and fauna. Furthermore, root system architecture plays a critical role in soil structure formation. Plants producing large quantities of fine roots appear to be more effective in soil aggregate formation compared to fibrous roots which can be less effective in fracturing soil aggregates^6^. The presence of roots increases aggregate stability, the permeability of soil^7^, soil porosity and pore connectivity^8^, and asserts a great influence on microbial community structure in terms of both richness and diversity^9,10^.

In agricultural systems, cover crops are being increasingly sown between main crops^11^ because they sequester carbon^3,12^, decrease soil erosion^3^, increase soil macro-porosity^13,14^, and increase microbial diversity and richness^15,16^. Cover crops are also associated with increased abundance of saprophytic and mycorrhizal fungi within the microbial community^10,17,18^. Most recent studies in this area have revealed that cover crops play a fundamental role in increasing the diversity and activity of the microbial community^9,16,18^. However, the effect of cover crops on soil structural genesis is poorly understood. Scott *et al*. have described the role of the aggregate sizes in the recovery of physically sequestered carbon^12^. Physical structure of soil was visualised using aggregate properties but there was no description of pore structure associated with this study. X-ray Computed Tomography (CT) can non-destructively image the soil pore networks and plant root morphology^14,19,20^. By using this technique, it was revealed that cover crops induced contrasting behaviour to changes in soil bulk density^14^. However, in this study, the focus was on root morphology, not soil structural properties. Amongst all these studies, the role of cover crops upon soil structural genesis was not described.

Our aim here was to assess the impact of four species of plants commonly grown as cover crops in the UK (white clover, black oat, phacelia, tillage radish) on soil structural genesis and modification of microbial community structure. These plants were selected for their contrasting root morphologies in terms of tap root formation, vigorous deep-rooting and fibrous multi-branching root systems. We hypothesised that different root morphologies would influence soil structural genesis dependent upon the root phenotype: for example, tap root species may initiate concentric compaction surrounding the primary root, decreasing porosity and diversity of pore sizes, compared to fibrous root species which may create a greater diversity of pore sizes, increasing porosity and pore-connectivity. X-ray CT was used to quantify the formation of pore networks in 3D and phospholipid fatty acid (PLFA) analysis was performed to study the microbial community phenotype.

## Results

### Characteristics of the pore architecture

**Figure 1:**
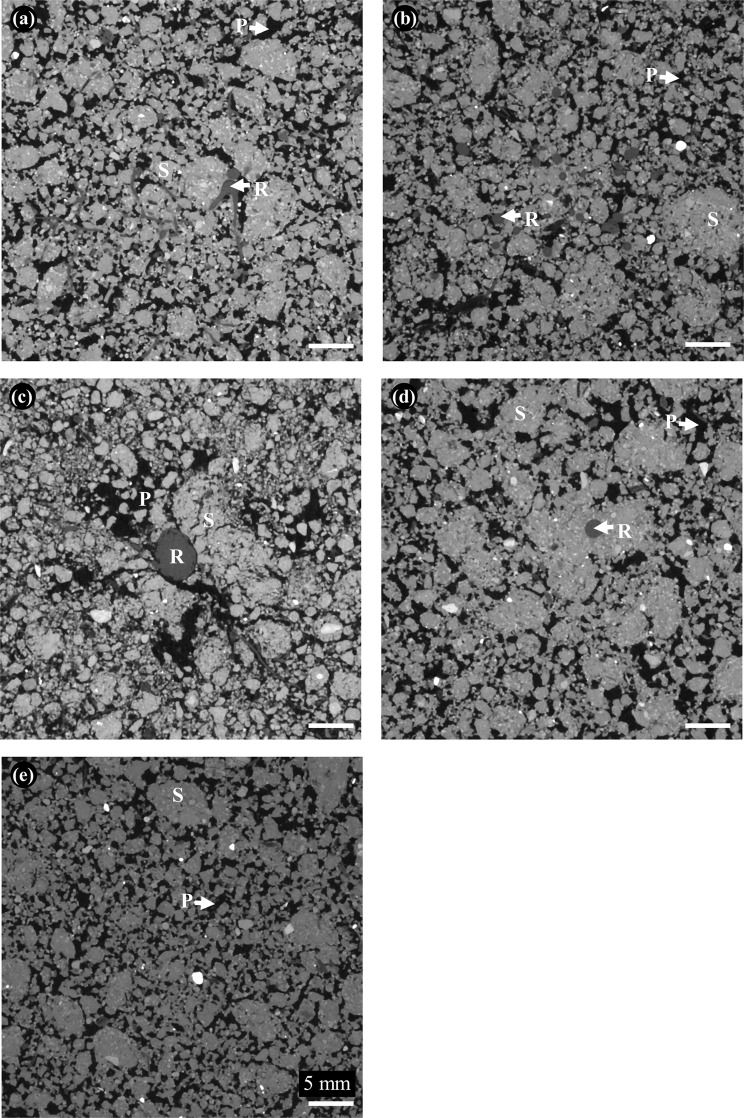
2D images of cores (40 μm resolution) at Week 8, displayed as greyscale images denoting X-ray attenuation (darker shades relate to lower attenuation), region of interest: (**a**) white clover; (**b**) black oat; (**c**) Phacelia; (**d**) tillage radish; and (**e**) unplanted soil (S: soil matrix, P: pore, R: root).

**Figure 2:**
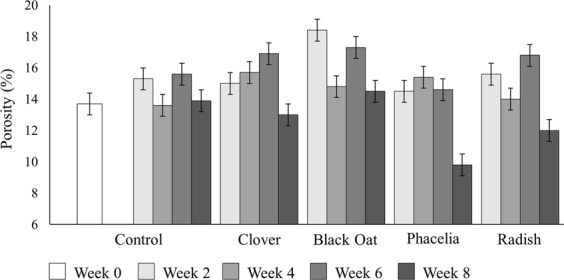
Porosity in relation to the planted treatment over the 8 weeks of growths expressed as percentage of relative pore to the total volume. Bars indicate means and whiskers denote pooled standard errors.

As hypothesized, the different plant species had contrasting effects on soil structure, apparent from visual inspection of the X-ray CT images (Fig. 1). For example, the roots of white clover, black oats and tillage radish appeared to grow through and around aggregates without forming cracks (Fig. 1a,b,d) compared to phacelia that had a wider root system which created cracks in the surrounding area (Fig. 1c). Formal quantification of soil structural parameters confirmed this. There was significant interaction between treatment and time with respect to porosity (*P* < 0.001; Fig. 2). Porosity of unplanted soil remained constant over the 8 weeks of plant growth with only slight increases after 2 and 6 weeks. In the presence of black oat, porosity increased significantly after 2 and 6 weeks, but was similar to unplanted soil at Weeks 4 and 8. For clover, phacelia and radish, the porosity was essentially constant up to 6 weeks and then decreased at Week 8 drastically (Fig. 2).

**Figure 3:**
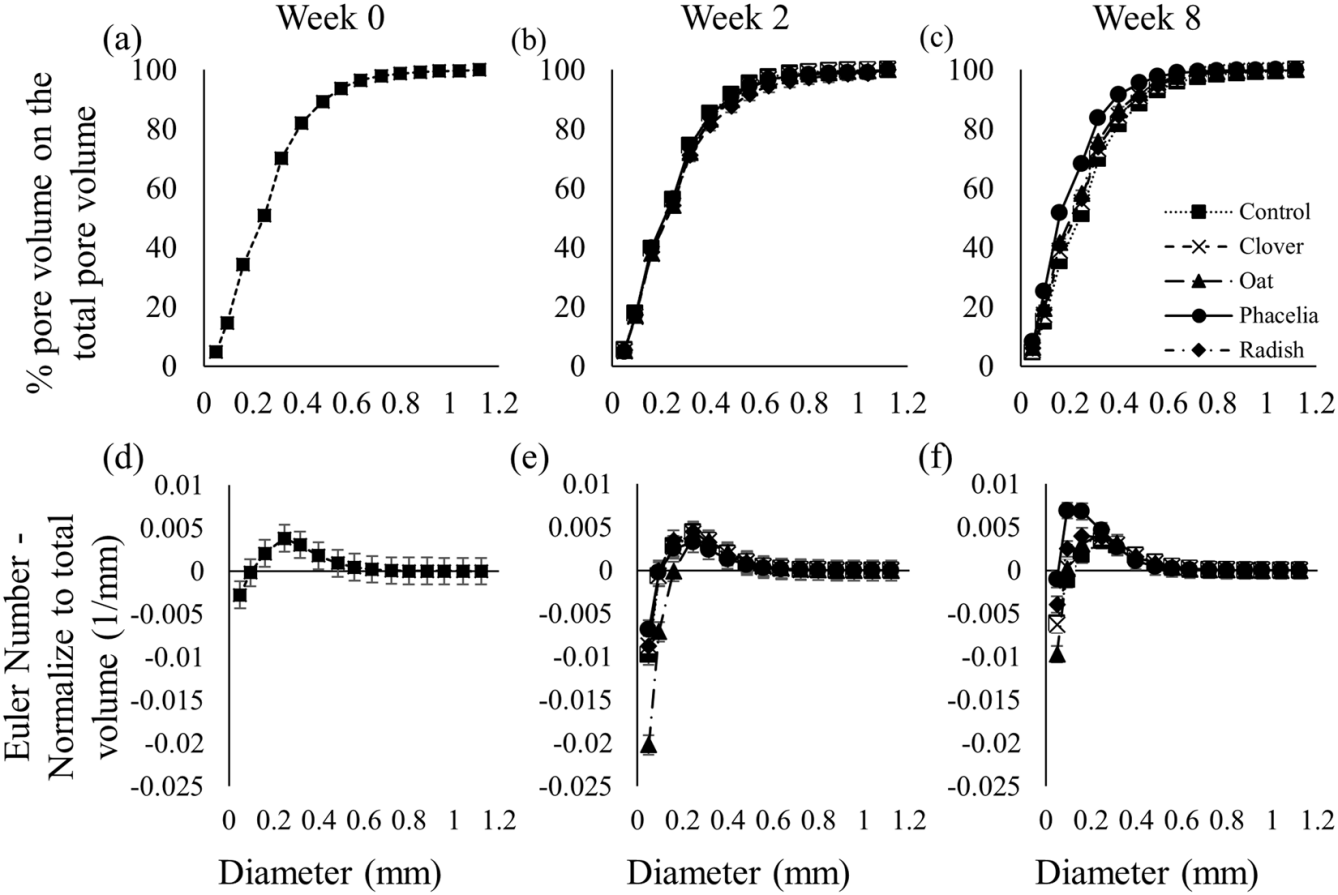
Minkowski functions of treatments at core scale (40 μm resolution) at three time points, week 0 (**a**,**d**) week 2 (**b**,**e**) and week 8 (**c**,**f**): (**a**–**c**) cumulative pore size distribution; (**d**–**f**) pore-connectivity of cores. Points show means, whiskers denote pooled standard errors. These fall within confines of points in some instances.

There were significant interactions between treatment and time with respect to cumulative pore size distribution at Week 8 (Fig. 3c), and pore-connectivity at Weeks 2 and 8 (Fig. 3e,f; *P* < 0.001). At Weeks 0 and 2, the pore size distribution was essentially congruent for all treatments with approximately 50% of pore sizes <0.25 mm and 80% of pore sizes <0.4 mm (Fig. 3a–c). At Week 8, phacelia increased the proportion of smaller pore sizes, with approximately 50% of pore sizes <0.16 mm and 80% of pore sizes <0.31 mm (Fig. 3c).

At Week 0, the unplanted columns had low pore connectivity evident from small Euler numbers for pores <0.09 mm (Fig. 3d). At Week 2, the overall pore connectivity had increased from Week 0. Soil planted with black oat had the greatest pore-connectivity, whilst the soil planted with phacelia was the least connected pore-system (Fig. 3e). At Week 8, the pore connectivity decreased for all treatments with the same pattern as Week 2: black oat soil had the greatest pore connectivity and phacelia the lowest pore-connectivity (Fig. 3f). There was a gradual change of pore size distribution and pore connectivity during the 8 weeks, and the data of Week 4 and 6 are shown in Supplementary Fig. 1 but omitted from Fig. 3 for clarity. At the aggregate scale, there were no significant differences in porosity, pore size distribution and pore connectivity between any of the treatments (Supplementary Fig. 2).

### Aggregate size distribution

**Figure 4:**
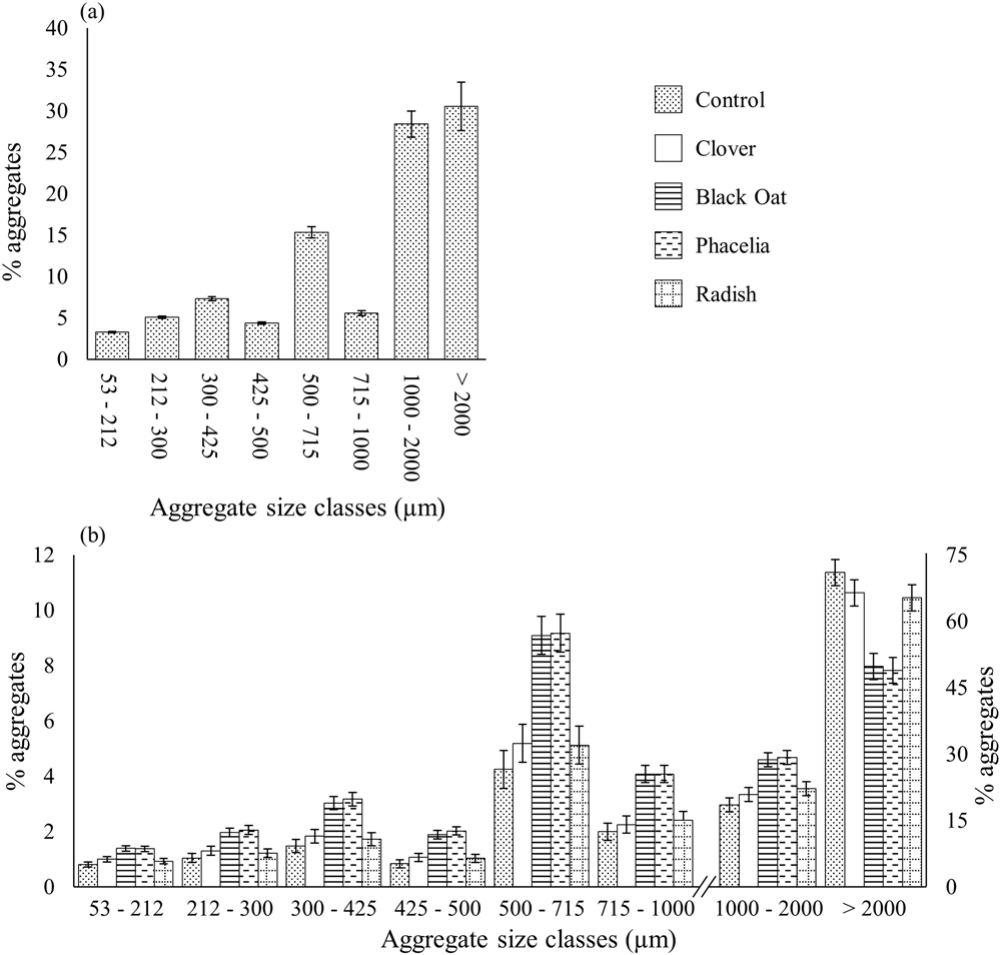
Aggregate size distribution of soils at Week 0 (**a**) and as influenced by different plant species after 8 weeks (**b**). Bars represent means expressed as percentage of aggregates relative to the total volume, and whiskers are pooled standard errors. Note difference in y-axis scale for size classes >1000 µm.

The aggregate size distribution was realised by a dry sieving of the soil through a series of sieves sized 2000, 1000, 850, 500, 425, 300, 212 and 53 µm. At Week 0, the aggregate size distribution of unplanted soil showed an increase in size. This trend was not consistent for the size classes (425–500 and 715–100 µm), where for these sizes there was a significantly smaller proportion of aggregates than neighbouring ones (Fig. 4a). This basic pattern persisted at Week 8 with a substantial increase in proportion of aggregates for the size class >2,000 µm. Planted soils with black oat and phacelia had a significantly greater proportion of aggregate between 2,000–300 µm than the unplanted soil and soil planted with clover and radish, but this trend was reversed for aggregates >2,000 µm (Fig. 4b).

### Microbial phenotypic profiles

**Figure 5:**
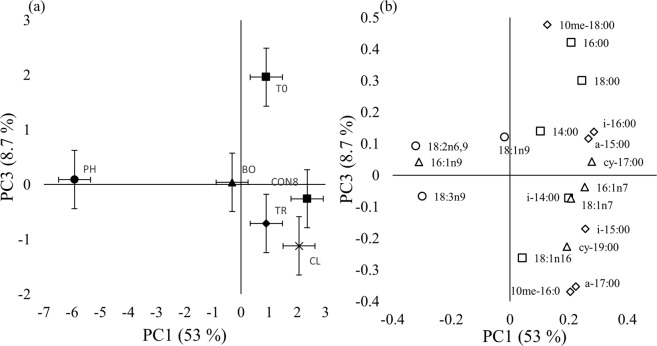
Effects of plant species upon microbial community phenotypes. (**a**) Principal component (PC) ordination of first and third PCs (T0: control at week 0, CON8: control at week 8, CL: white clover, BO: black oat, PH: Phacelia, TR: tillage radish; points show means, whiskers denote pooled standard errors) and (**b**) associated loading (◊ Gram positive ∆ Gram negative ○ fungi □ non-specific markers).

There were significant plant effects upon microbial community phenotypic structure with respect to PC1 and PC3 (both *P* < 0.001), which collectively accounted for 61% of the variation (Fig. 5). Microbial community phenotype profiles differed significantly between both planted and unplanted soils, as well as between plant species. There was a significant effect of plant species upon the microbial community phenotype apparent via PC1 and PC3 (*P* < 0.001 and *P* = 0.012 respectively) which together accounted for 61% of the variance. Community structure associated with phacelia was notably distinct from the other treatments apparent via PC1, and communities associated with black oat distinct from those of clover, with black oat communities intermediate between these (Fig. 5a). PC3 discriminated communities associated at Week 0 (control) with those present at Week 8 in all cases (Fig. 5a). The loadings associated with PC1 were predominantly two fungal biomarkers (18:2n6,9; 18:3n9) and two Gram-negative biomarker (16:1n9; cy-17:00) and two Gram positive markers (i-16:00; a-15:00; Fig. 5b). Markers associated with Gram-positive bacteria contributed large loadings to PC3 (Fig. 5b).

**Figure 6:**
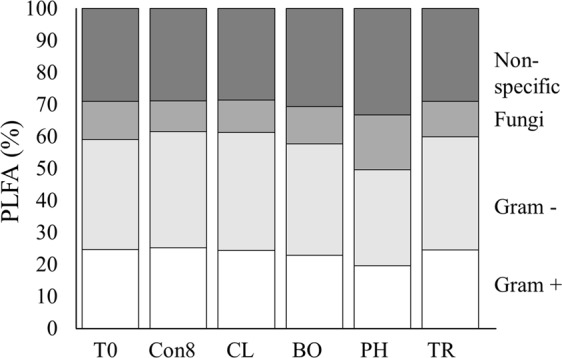
Proportion of PLFA divided per group of community: T0: control at week 0; after 8 weeks of incubation: Con8: control; CL: clover; BO: Black oat; PH: phacelia and TR: Tillage radish.

The nature of the effect was due to an increase of the proportion of fungal markers and a decrease in the proportion of Gram positive bacterial markers for phacelia compared to the other treatments (Fig. 6).

### Root growth

After 6 weeks of growth, there were marked visual differences in the morphology of the root systems of the different species, with black oat and phacelia showing substantial extensive growth throughout the soil volume, in contrast to clover which showed sparse growth, particularly below the upper 2 cm of the soil column (Supplementary Fig. 3a–c). Tillage radish showed a strong vertical development of the primary root, with intermediate exploitation of the soil volume via lateral root growth (Supplementary Fig. 3a–c).

## Discussion

The apparently limited effects on soil structure of white clover and tillage radish might have been due to a lesser development of root systems, as both plant species grew much more slowly than black oat and phacelia (Supplementary Fig. 3). After 8 weeks, the increase in proportion of the largest aggregates (>2,000 µm) for the control, the white clover and tillage radish might be caused by the high concentration of clay particles^4,21,22^. For black oat and phacelia treatments, the presence of plant roots decreased the proportion of the largest aggregates (>2,000 µm) by maintaining a greater proportion of aggregate sizes of 1,000–2,000 µm compared to the control. Therefore, the high proportion of roots decreased the proportion of larger aggregates (>2,000 µm), and increased the proportion of aggregate sizes from 1,000–2,000 µm (Fig. 4). The greater presence of roots in black oat and phacelia columns could have induced the breakdown of the larger aggregates, i.e. penetration roots through existing pores can destabilise macro-aggregate resulting in an increase in proportion of aggregate sizes from 2,000–1,000 µm^5,6^.

The growth of black oats did not significantly alter soil porosity (Fig. 2). Between Weeks 2 and 8, black oat decreased pore connectivity (Fig. 3b,c). However, for Weeks 2 and 8, black oat had the greatest pore connectivity of all treatments (Fig. 3b,c). Black oats maintained a greater porosity and pore-connectivity in contrast to phacelia which significantly decreased both porosity and pore-connectivity at Week 8 (Figs 2 and 3c) and increased the proportion of smaller pores compared to all treatments at Week 8 (Fig. 3f). This apparent contradiction of effects on aggregate size distribution and 3D pore characteristics might be due to the fact that both plant species influence soil structure via modification of aggregates. Re-arrangement of aggregates might be different depending on the root morphologies as black oat has generally thicker and more structured root architecture compared to phacelia which has thinner roots with less organized structure^14,23^. This formation of pore sizes (between 0.05 to 0.16 mm) facilitates flow of water, gas and nutrients^19^, however, the decrease in pore connectivity might counteract this shift in pore sizes^24,25^. Thus, the formation of new pores, smaller than 0.3 mm, with a poor-connection might be involved in the creation of water storage pores^26,27^. At Week 8, black oat and phacelia columns were root bound, notwithstanding that the nature of inherent pore network was drastically different for both these treatments. Root biomass could not be determined since aggregate sampling precluded this. Thus, different modifications of pore networks were implemented by the inherent nature of root systems and not by the extent of rooting. Therefore, after 8 weeks of growth, black oat and phacelia showed evidence of impacting the pore network differently by modifying its characteristics in very different ways.

The lack of significant influence of the plant at aggregate scale (Supplementary Fig. 2) might be influenced by the water regime, which was kept constant during the experiment. Wet and dry cycles are important for modification of soil structure^26^. Notably by the disruption of aggregation due to clay particles swelling in the presence of water and the compression of trapped air in capillary pores which could lead to the creation of new pores^28,29^. Here, a soil survey has characterised the clay as containing kaolinite and vermiculite, which has respectively a low and a moderate shrink swell capacity^30^. The presence of plants imposes localized wet-dry extremes at the immediate root surface leading to greater cohesion of root exudates and clay particles^31,32^. The lack of significant effects of plants on the micro-structure might be due to an absence of wet-dry extremes and the mineralogy properties.

Principal component analysis (PCA) discriminated the microbial community at Week 0 compared to all the treatments at Week 8 via PC3: microbial community phenotype changed over the incubation period via PC3, but the species or control at Week 8 were not discriminated (Fig. 5a). This discrimination was associated with a shift of the bacterial community between both time points (Fig. 5b). Notwithstanding this, PCA distinctly discriminated phacelia in relation to PC1, which was associated with the saprophytic fungal biomarkers 18:2n3,6 and 18:3n9 and Gram-negative bacteria 16:1n9 (Fig. 5)^33–36^. Moreover, phacelia showed a greater proportion of fungal biomarkers compared to all treatments which revealed that phacelia increased the presence of saprophytic fungi (Fig. 6). Such microbes were likely utilising rhizodeposits as Gram-negative bacteria and fungi have been described to be involved in rapid assimilation of rhizodeposit carbon in grassland soils^36,37^. Another study showed that approximately two months after sowing, fungi were an important factor, especially the non-mycorrhizal fungi, to discriminate microbial community structure between different cover crops^18^. Phacelia has been described as forming mycorrhizal associations^38–40^, but there is currently no apparent record in UK soils of mycorrhizal formation. A quantification of the mycorrhizal infection was performed on the phacelia root (Supplementary Material and Methods 2), which revealed no colonisation of roots by mycorrhizal hyphae (Supplementary Fig. 4). The discrimination of black oat and tillage radish via PC1 between control and clover treatments (Fig. 5a), showing that both plant species slightly impacted microbial community structure but not to the same extent as phacelia, which could be due to the nature of root characteristics^41^.

Furthermore, differences in microbial community structure can have a significant impact on soil physical structure. For example, fungal dominated communities increase porosity at a scale relevant to water storage and flow and gas exchange^42,43^. The nature of the carbon input can modulate the microbial impact on soil structure via contributing or hindering crack formation. This revealed the role of the microorganisms as degraders or as producers of soil binding agents^44^. Black oat and phacelia had opposite impacts on the soil (Fig. 3) and enhanced different microbial community phenotypes, which raises the hypothesis that the microbial community can contribute to the modification of the soil structural genesis as well as the plant root morphologies.

## Methods

### Preparation of soil columns

Soil from the Worcester series, a clay soil (clay: 43.3%, silt: 28.4%, sand: 28.3%) determined from the method of^45,46^, was collected from 0–50 cm depth from an arable field situated at the University of Nottingham experimental farm in Bunny, Nottinghamshire, UK (52.52°N, 1.07°W). The soil was air-dried for 2 days, and passed through a 2 mm mesh sieve, to provide a homogenised structure. To re-activate the microbial community, the soil was re-wetted to 15% moisture content and incubated in bulk in black plastic bags slightly opened in a dark room at room temperature and then passed through a 10 mm sieve to ensure effective homogenisation. Polypropylene tubes (170 mm height × 68 mm diameter) with a 0.1 mm mesh adhered to the bottom were packed with the moist soil to a bulk density of 1 g cm^−3^. Columns were saturated for 24 h via capillary rise, and left to drain for 48 h to reach field moisture capacity (20% ± 1%). Four cover crop species were selected for their contrasting root morphologies: a tap root species, tillage radish (*Raphanus sativus* L. cv. “Mimo”), a vigorous deep-rooting species, black oat (*Avena strigosa* L. cv. “Prate”), and two fibrous multi-branching species, white clover (*Trifolium repens* L. cv. “Galway”) and phacelia (*Phacelia tanacetifolia* Benth. cv. “Angelia”). These species are commonly used as cover crops in arable systems. One pre-germinated seed was sown per individual columns and adjusted to contain one emergent plant per column. Twenty replicates of each plant species, and of an unplanted (control) soil, were allocated in a random block design to allow for four replicates of each treatment to be sampled at Week 0, 2, 4, 6 and 8. Columns were maintained in a growth chamber set at 16:8 h light:dark cycle, 21:15 °C respectively and 70% humidity. The moisture content was kept constant during the experiment by watering the plants every day controlled by the weight of the columns.

### X-ray computed tomography (CT)

Homogenisation of column packing was checked by X-ray CT. Planted and unplanted columns were scanned using Phoenix v | tome | x M scanner (GE Measurement and Control solution, Wunstorf, Germany) set at a voltage of 180 kV with a current of 180 μA and at voxel resolution of 40 μm. A multiple scan was performed for 1 h 29 s, with a total of 2160 projection images taken at a 250 ms period using an averaging of 3 images and a skip of one. Longer scanning than might be routinely employed was favoured to obtain the enhanced image contrast for thresholding. Columns were destructively harvested after being scanned, and from the air-dried soil, three aggregates were randomly selected per column (Supplementary Materials and Methods 1).

Scanned images were reconstructed using Phoenix datos | x2 rec reconstruction software. The scanned images were optimised to correct for any sample movement during the scan and to reduce potential noise using the beam hardening correction algorithm, set at 8. As a multi-scan routine was performed on the soil column samples, VG StudioMax^®^ 2.2 was used to merge the top, middle and bottom scans to obtain a single 3D volume for the complete column. Image sequences of 40 × 40 × 120 mm were extracted for image analysis from the columns.

### Image analysis

Image manipulation was performed using Image J^47^. This step consisted of cropped the image sequence, applied a median filter (averaging 2 pixel), enhanced brightness and contrast and selected 2 threshold values. The segmentation and quantification of 3D pore characteristics was processed using QuantIm^48^ following a standard method^8^, which is explained briefly here. The threshold of the pore system was realised on the 3D volume, and included only the pores, the root materials was comprised in the solid phase. This step was facilitated due to the long scanning procedure which created a better image quality. The quantification of the pores was quantified in 3D using QuantIm. The 3D characteristics were: (i) percentage of pores with a size greater than the scanning resolution (40 µm), hereafter referred as porosity; (ii) pore size distribution, *viz*. the proportion of each pore size class within the range 0.05–1.1 mm (for the columns) normalised by the total pore volume, expressed as a cumulative value; (iii) pore-connectivity, determined by the Euler number normalized to the total volume^48^: the more negative the Euler number, the greater the pore-connectivity.

### Sampling and measurement

Every two weeks (0–8), 4 replicates per treatments were scanned as above and then destructively harvested. Subsamples (c. 20 g) of the moist soil were stored at −82 °C and then freeze-dried; the rest of the soil was air-dried for further analysis. The freeze-dried and air-dried soils were stored in the dark at room temperature.

### Aggregate size distribution

Aggregate size distributions were determined by passing 250 g of air-dried soil through a series of sieves sized 2000, 1000, 850, 500, 425, 300, 212 and 53 µm, via horizontal shaking for 3 minutes at 300 rotation.min^−1^ on a horizontal KS 500 shaker (Janke & Kunkel, Staufen, Germany)^49^. The mass of soil retained on each sieve was determined and normalized by the total mass of the sieved soil^50^.

### Microbial community phenotype profiling

The microbial community phenotypic community structure was profiled using the PLFA technique^51^. PLFA were extracted from 2 g of freeze-dried soil following a method derived from^51,52^. The lipid classes were separated using a solid phase extraction (SPE) column using Hypersep SPE column containing 50 mg of silica per 1 mL column. The extracted lipids were methylated via a transesterification process to convert them into dried fatty acid methyl ester. The fatty acids were dissolved in 75 µL of hexane, for gas chromatography (GC) analysis. The GC analysis was performed using a GC and a DSQII mass spectrometer (Thermo Electron Corporation^®^), equipped with a Zebron capillary ‘ZB-FFAP’ column from Phenomex^®^. The dimensions of the column were 30 m length × 0.25 mm inner diameter × 0.25 µm film thickness. The method was 1 µL of the sample was injected in the column maintained at a constant temperature of 250 °C, the carrier gas was helium set at 12.4 × 10^4^ Pa. For each sample, a chromatogram was obtained with the retention time of each compound and the ion profile provided by the mass spectroscopy.

The markers were associated to different microbial groups as follows: Gram positives: i-15:00, a-15:00, i-16:00, a-17:00, 10me-16:00, 10me-18:00; Gram negatives: 16:1n9, 16:1n7, cy17:00, 18:1n7, cy19:00; saprophytic fungi: 18:1n9, 18:2n6,9, 18:3n9; and non-specific: 14:00, i-14:00, 16:00, 18:00, 18:1n16^33–35,53^. The percentage of the fatty acid indicators was used to analyse the proportion of microbial groups. The proportion of the microbial groups were calculated by the sum of all markers.

### Statistical analysis

For the pore characteristics, two-way analysis of variance (ANOVA) was conducted using Genstat v 17.1 (VSN International Ltd 2014), performed on all primary variables using a split-plot design with the plant treatments and size classes of pores as factors and for the total porosity, time was added to the factor list. For the aggregate size distribution data a separate ANOVA was performed for each size class due to order-of-magnitude differences in values between classes. PLFA profiles were analysed by principal component (PC) analysis and resultant PCs analysed by ANOVA.

## Conclusions

These results revealed a contrasting effect of root morphologies on soil structural genesis, which validated our hypothesis. Vigorous deep-rooting species (represented by the black oats) maintained the porosity and pore connectivity whereas one of the fibrous multi-branching root species (phacelia) decreased porosity and pore-connectivity and enhanced the proportion of smaller pore (<0.31 mm). The tap root species (represented by tillage radish) and the second species of the fibrous multi-branching root species (white clover) decreased the porosity but had no significant impact on the pore connectivity. Therefore, the nature of the root architecture of these plant species likely modified the soil pore characteristics differently depending on the growth strategy of the plants. Moreover, the microbial community phenotype was also modified by the presence of plants.

These results confirmed that the diversity of root morphology and higher-order interactions between plant and soil biota impact soil structural genesis and dynamics^54^. This has practical and ecological implications since the nature of root morphology can have different effects upon soil structure. What is unclear is the extent to which such effects occur where plants are growing in combination, which occurs in natural systems, and can be prescribed in cover-crop mixtures. This warrants further investigation.

## Supplementary information

Supplementary material & methode and figures
