# Supplementary material for: Cover crop species have contrasting influence upon soil structural genesis and microbial community phenotype
Source: Sci Rep. 2019 May 16;9:7473. doi: 10.1038/s41598-019-43937-6 (PMC6522496; doi:10.1038/s41598-019-43937-6)
Supplement: Supplementary file 1 — Supplementary material & methode and figures [file 41598_2019_43937_MOESM1_ESM.pdf]

Appendices

Cover crop species have contrasting influence upon soil structural genesis and microbial community phenotype

Aurelie Bacq-Labreuil, John Crawford, Sacha J. Mooney, Andrew L. Neal, Karl Ritz

Supplementary Methods 1. Aggregate extraction and image analysis

Supplementary Methods 2. Root segmentation

Supplementary Methods 3. Clearing, staining and quantifying mycorrhizal roots

Supplementary Fig. 1: Minkowski functions of treatments at core scale (40 µm resolution) at two time points, Week 4 (a, c) and Week 6 (b, d): (a, b) cumulative pore size distribution; (c, d) pore-connectivity of cores. Points show means, whiskers denote pooled s.e.

Supplementary Fig. 2: Minkowski functions of treatments at aggregate scale (1.5 µm resolution) at two time points, Week 0 (a, b, d) and Week 8 (a, c, e): (a) porosity (b, c) cumulative pore size distribution; (d, e) pore-connectivity of cores. Bar chart and points show means, whiskers denote pooled s.e.

Supplementary Fig. 3: An example of segmented roots at Week 6 from (a) white clover, (b) black oats, (c) phacelia and (d) tillage radish. Scale bar is 2 cm.

Supplementary Fig. 4: An example of images visualised with a binocular microscope from the phacelia (a) unstained roots; (b) stained roots. Scale bars are 500 µm.

Supplementary Methods 1. Aggregate extraction and image analysis

Aggregates were selected randomly from the sieve 2000-1000 μm and scanned using Phoenix Nanotom® (GE Measurement and Control solution, Wunstorf, Germany) set at a voltage of 90 kV, a current of 65 μA and at a voxel resolution of 1.51 μm. The total scan time was 69 minutes, with a total of 1440 projection images was taken at 500 ms period using an averaging of 3 images and skip of 2. Scanned images were reconstructed using Phoenix datos|x2 rec reconstruction software. The scanned images were optimised to correct any sample movement during the scan and reduce noise using the beam hardening correction algorithm, set at 8. Image sequences of 0.98 x 0.73 x 0.60 mm were extracted for the image analysis for the aggregates. The image analysis was performed exactly as the core images.

Supplementary Methods 2. Root segmentation

The root segmentation was processed using the plugin Root1 in Image J[1]. The threshold values were selected manually in Image J. There was no filter or image enhancement applied. To clean-up the segmented roots, the image sequences were imported in Volume Graphic Studio MAx 2.2. All materials were selected using a surface determination of the binary output from image J to create an imitial region of interest. Then all connected roots attached to the stem were selected out using the region growing tool to create a root ROI. Noise within the data was deleted with the eraser tool. The 3D rendering was visualised with VG Studio Max 2.2.

Supplementary Methods 3. Clearing, staining and quantifying mycorrhizal roots.

The quantification of mycorrhizal infection of roots was performed on fresh root samples harvested at the end of the experiment. Clearing and staining of roots was proceeded following the method from[2]. All the baths were kept at 70ºC for the experiment. Roots were

cleared in 10% potassium hydroxide solution for 90 mins, then washed for 2 mins in water. Roots were stained in 3% ink in 5% acetic acid solution for 6 mins and washed in 5% acetic acid for 10 mins. Roots were placed in Petri dish with gridlines containing 50% of glycerol, the quantification of the mycorrhizal infection was performed following the method from[3] using a binocular microscope (Stemi SV 6, Zeiss, Germany), and an associated camera (Axioma ERc 5S, Zeiss, Germany) using the in-build software Zen® 2.3 lite.

References:

1. Flavel, R. J., Guppy, C. N., Rabbi, S. M. R. & Young, I. M. An image processing and analysis tool for identifying and analysing complex plant root systems in 3D soil using non-destructive analysis: Root1. *PLoS One* **12,** 1–18 (2017).
2. Vierheilig, H., Coughlan, A. P., Wyss, U. & Piche, Y. Ink and Vinegar, a Simple Staining Technique for Arbuscular-Mycorrhizal Fungi. *Appl. Environ. Microbiol.* **64,** 5004–50070 (1998).
3. Giovannetti, M. & Mosse, B. An evaluation of techniques for measuring vesicular arbuscular mycorrhizal infection in roots. *New Phytologist* **84,** 489–500 (1980).

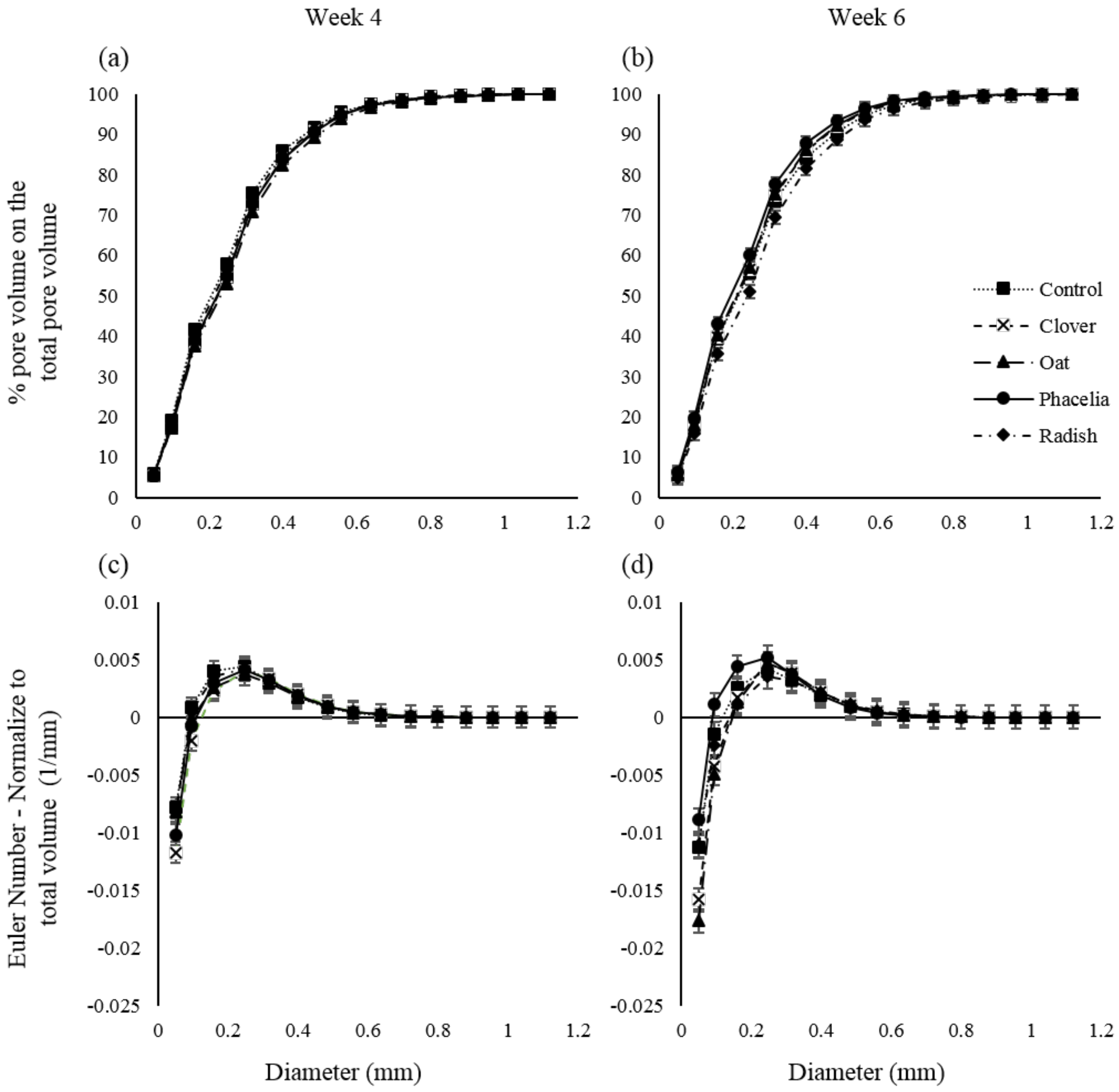

Supplementary Fig. 1: Minkowski functions of treatments at core scale (40 µm resolution) at two time points, Week 4 (a, c) and Week 6 (b, d): (a, b) cumulative pore size distribution; (c, d) pore-connectivity of cores. Points show means, whiskers denote pooled s.e.

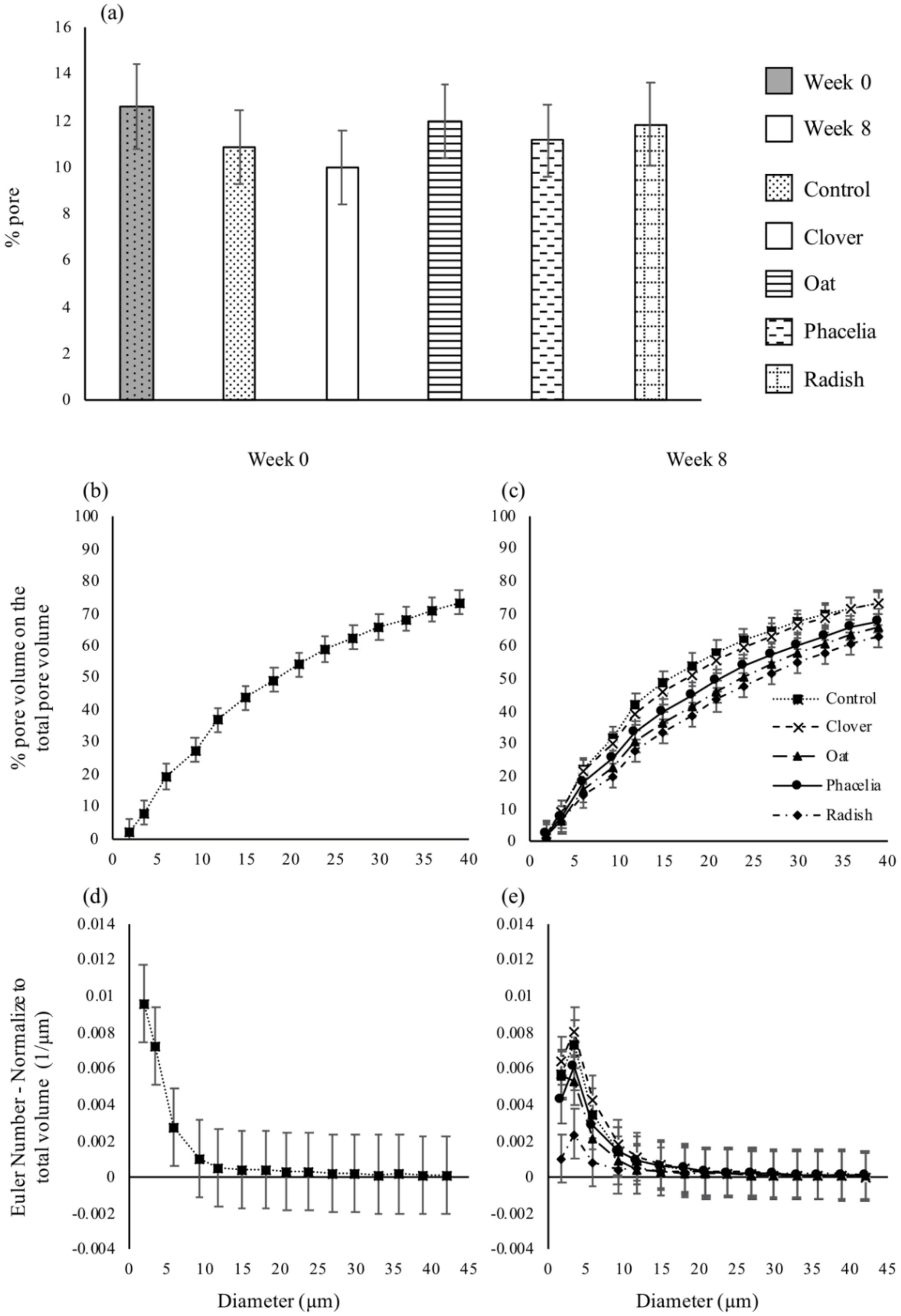

Supplementary Fig. 2: Minkowski functions of treatments at aggregate scale (1.5 μm resolution) at two time points, Week 0 (a, b, d) and Week 8 (a, c, e): (a) porosity (b, c) cumulative pore size distribution; (d, e) pore-connectivity of cores. Bar chart and points show means, whiskers denote pooled s.e.

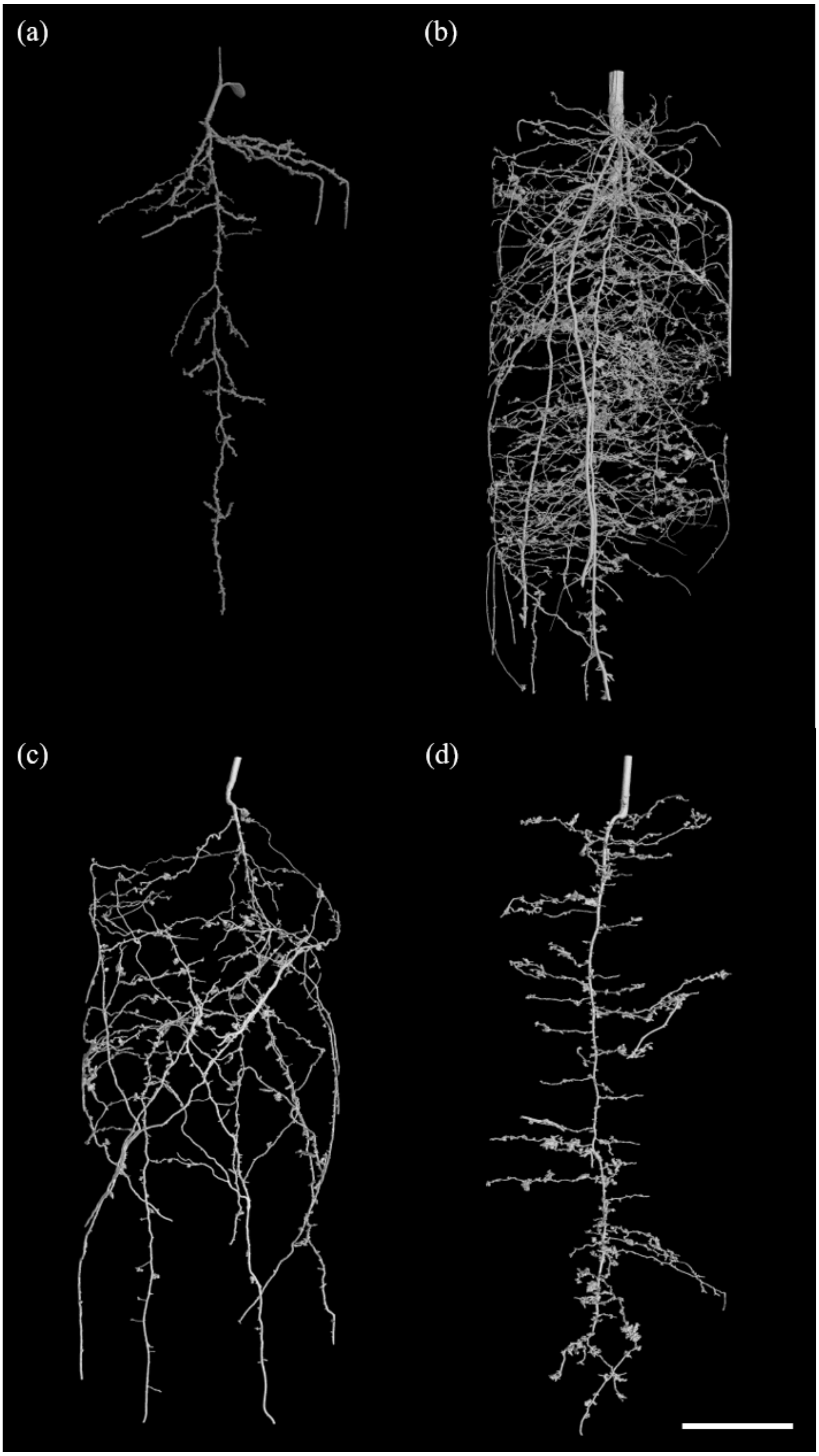

Supplementary Fig. 3: An example of segmented roots at Week 6 from (a) white clover, (b) black oats, (c) phacelia and (d) tillage radish. Scale bar is 2 cm.

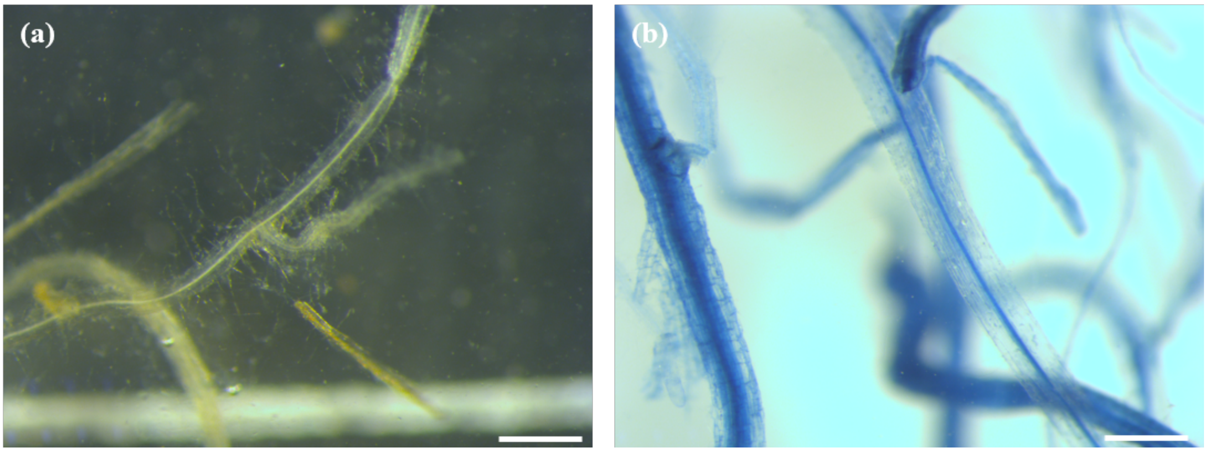

Supplementary Fig. 4: An example of images visualised with a binocular microscope from the phacelia (a) unstained roots; (b) stained roots. Scale bars are 500 µm.
